# Lagrangian formulation of Omori’s law and analogy with the cosmic Big Rip

**DOI:** 10.1140/epjc/s10052-020-8019-2

**Published:** 2020-05-19

**Authors:** Valerio Faraoni

**Affiliations:** Department of Physics and Astronomy, Bishop’s University, 2600 College Street, Sherbrooke, QC J1M 1Z7 Canada

## Abstract

A recent model predicting Omori’s law giving the number of aftershocks per unit time following an earthquake involves a differential equation analogous to the Friedmann equation of cosmology. The beforeshock phase is analogous to an accelerating universe approaching a Big Rip, the main shock to the Big Rip singularity, and the aftershock to a contracting universe. The analogy provides some physical intuition and Lagrangian and Hamiltonian formulations for Omori’s law and its generalizations.

## Introduction

One of the first results obtained in modern seismology was Omori’s law stating that, on average, following a strong earthquake the number of aftershocks per unit time *n*(*t*) decays according to the empirical power law^1^ [1]

1 $$\begin{aligned} n(t)=\frac{k}{c+t}=\frac{k}{t-|c|} \,, \end{aligned}$$

where $k>0$ and $c<0$ are constants. There is a large body of literature on Omori’s law (see [4, 5] for reviews), but its physical interpretation is still mysterious, although it seems clear that somehow the source of the earthquakes should be traced to a rupture mechanism in the rocks composing the Earth’s crust. There is some belief that Omori’s law is fundamental and not a mere data-fitting device and, in this optics, it makes sense to derive it from basic models.

The derivation proposed in Refs. [5–7] begins by noting that *n*(*t*) satisfies the first order differential equation

2 $$\begin{aligned} \dot{n}=-\sigma \, n^2 \,, \end{aligned}$$

where $\sigma =k^{-1}$ and an overdot denotes differentiation with respect to time. The derivation uses an analogy between the decaying number of aftershocks per unit time and the decreasing density of ionospheric plasma due to the recombination of opposite charges [5–7]. If $n_{\pm }$ is the density of positive/negative charges and $n=n_{+}+n_{-}$, the recombination equation becomes $\dot{n}=-\sigma n_{+} n_{-}$ and approximates to Eq. (2) for a globally neutral plasma in which $n_{+} \simeq n_{-}$. Similarly, an earthquake occurs due the fast slip of rock along a fault plane in the Earth’s crust, and there are two adjacent sides (denoted with $n_{+}$ and $n_{-}$) of a tectonic fault. Rupture releases the energy in an active fault and neutralizes the stresses on the parallel sides of it, reducing the number *n* of active faults. The evolution of the number of faults then should obey [5–7]

3 $$\begin{aligned} \frac{dn}{dt} = -\sigma n_{+} n_{-} \simeq -\sigma n^2 \end{aligned}$$

where $\sigma$ is a deactivation coefficient and $n_{+}=n_{-}$ has been used. The fact that a pair of adjacent fault sides is involved rules out different powers in the Omori law^2^ [5, 7].

The beforeshock phase, during which secondary shocks increase their frequency until the main shock, can be described by a version of Omori’s law $\dot{n}= \sigma \, n^2$, although the phenomenological descriptions and data fitting are different. Here we point out that there are many similarities between the differential equation satisfied by Omori’s law and the Friedmann equation of spatially homogeneous and isotropic Friedmann–Lemaître–Robertson–Walker (FLRW) cosmology [8–13]. The analogy holds in the case of a universe with a phantom fluid as the matter source and with a Big Rip singularity occurring at a finite time. The Big Rip separates the “before” and “after” universes and is analogous to the main earthquake shock. This analogy is intriguing and may provide some physical intuition about variability of the deactivation coefficient $\sigma$ versus variability of the power in Omori’s law. What is more, the analogy reveals previously unknown Lagrangian and Hamiltonian formulations of the physical system described by the Omori law (2) and its generalizations.

In the next section we discuss the Lagrangian and Hamiltonian associated with the Omori law (2). In Sect. 3 we recall the basics of FLRW cosmology and we present the analogy with a Big Rip in a spatially flat universe, while Sect. 4 contains the conclusions.

## Lagrangian formulation of Omori’s law and a mechanical analogy

It is not obvious that Omori’s law can be described using the Lagrangian or Hamiltonian formalisms. A Lagrangian leading to Omori’s law is

4 $$\begin{aligned} L\left( n, \dot{n} \right) = n\dot{n}^2 + \sigma ^2 \, n^5 \,. \end{aligned}$$

In fact, the Euler–Lagrange equation

5 $$\begin{aligned} \frac{d}{dt} \left( \frac{\partial L}{\partial \dot{n}} \right) -\frac{\partial L}{\partial n}=0 \end{aligned}$$

yields

6 $$\begin{aligned} 2n\ddot{n} +\dot{n}^2 -5\sigma ^2 n^4 =0 \,. \end{aligned}$$

Now, the Omori law (2) is a first integral of Eq. (6). In fact, by differentiating (2) one obtains

7 $$\begin{aligned} \ddot{n}=-2\sigma \, n \dot{n}=2\sigma ^2 \, n^3 \,, \end{aligned}$$

using which one verifies that $2n\ddot{n}+\dot{n}^2-5\sigma ^2 n^4 =0$.

The corresponding Hamiltonian is

8 $$\begin{aligned} {\mathcal {H}} = \pi _n \dot{n}-L = n \left( \dot{n}^2 -\sigma ^2 n^4 \right) \,, \end{aligned}$$

where $\pi _n \equiv \partial L/\partial \dot{n} = 2n\dot{n}$ is the momentum canonically conjugated to the variable *n*. One notes that $\partial {\mathcal {H}}/\partial t$ vanishes and the Hamiltonian is conserved, ${\mathcal {H}}=$ const. Furthermore, using the Omori law (2) in Eq. (8) gives

9 $$\begin{aligned} {\mathcal {H}}=0 \,, \end{aligned}$$

i.e., the point-particle system associated with the Omori Lagrangian and Hamiltonian has conserved total energy equal to zero.

One can write

10 $$\begin{aligned} \frac{\mathcal {H}}{2} = \mu \left( \frac{\dot{n}^2}{2} - \frac{\sigma ^2}{2} \, n^4 \right) \end{aligned}$$

where, for $n \ge 0$, $\mu (n)=n$ is a position-dependent mass, with kinetic energy $\mu \, \dot{n}^2/2$, potential energy $V(n)=-\mu \sigma ^2 n^4/2$, and zero total mechanical energy. Since $\dot{n}<0$, the particle will move to the left of the *n*-axis, tending toward $n=0$ (i.e., the seismic activity is more intense at the initial point $n_{(0)}>0$ and stops at $n=0$).

The $\left( n , \dot{n} \right)$ phase plane associated with Omori’s law has a very simple structure. Equation (2) or, equivalently, Eq. (9) is an energy constraint that reduces the orbits of the solutions to move on the parabolas $\dot{n}(n) = \mp \sigma n^2$, with the upper sign corresponding to the aftershock phase and the lower one to the beforeshock phase. The two parabolas correspond to the orbits of two different dynamical systems and are considered here as living in the same phase plane only for convenience: the fact that they touch each other at the origin (0, 0) has no meaning since these are disconnected curves.

The aftershock phase corresponds to the lower quadrant $n \ge 0, \dot{n}\le 0$, in which the point representing the state of the system moves along the downward-facing parabola towards the origin, which is an attractor. In this regime, secondary shocks decay in a finite time |*c*|.

The beforeshock phase corresponds to the upper quadrant $n\ge 0, \dot{n}\ge 0$, in which the point representing the dynamical system moves away from the origin and upward toward infinite *n* and $\dot{n}$, reaching infinity in a finite time. The main shock corresponds to infinity in this plane, to the pole $t=|c|$ in the solution

11 $$\begin{aligned} n(t)=\frac{k}{ \left| t -|c|\right| } \,, \end{aligned}$$

and to a discontinuity in the dynamics.

## Analogy with a cosmic Big Rip singularity

One can square Eq. (2) and rewrite it as

12 $$\begin{aligned} \left( \frac{\dot{n}}{n}\right) ^2 = \sigma ^2 n^2 \end{aligned}$$

which is analogous to the Friedmann equation of cosmology if one exchanges *n*(*t*) with the cosmic scale factor. In order to develop the analogy, let us recall the basics of FLRW cosmology [9–13].

In general relativity [8–10], a spatially homogeneous and isotropic universe can only have one of three possible geometries, which are described by the four-dimensional FLRW line element given, in comoving polar coordinates $\left( t, r, \theta , \varphi \right)$, by

13 $$\begin{aligned} ds^2 = -dt^2 +a^2(t) \left[ \frac{dr^2}{1-Kr^2} +r^2 \left( d\theta ^2 + \sin ^2 \theta \, d\varphi ^2 \right) \right] \,. \end{aligned}$$

The function *a*(*t*) (“scale factor”) quantifies how two points at fixed comoving distance $$r_0$$ (*e.g.*, two average galaxies without proper motions) move away from each other as the universe expands. Their physical separation at time *t* is $$ l(t)=a(t)r_0$$ and it increases in an expanding universe described by increasing *a*(*t*). Therefore, the scale factor *a*(*t*) illustrates the expansion history of the universe.

The constant *K* in Eq. (13) is normalized to $K= 1, 0, -1$ corresponding, respectively, to a closed universe (closed three-dimensional spatial sections $t=$ const.), Euclidean spatial sections, or hyperbolic 3-spaces [8–13], which includes all the possible FLRW geometries. The cosmic dynamics is described by *a*(*t*) [8–13].

In relativistic cosmology the matter content of the universe, which is the source of the spacetime curvature, is usually modeled by a perfect fluid with energy density $\rho (t)$ and isotropic pressure *P*(*t*). These quantities are related by some equation of state, usually (but not necessarily) of the form $P=w\rho$ with $w=$ const.

The functions $a(t), \rho (t)$, and *P*(*t*) obey the Einstein-Friedmann equations

14 $$\begin{aligned}&H^2 \equiv \left( \frac{\dot{a}}{a}\right) ^2 =\frac{8\pi G}{3} \, \rho -\frac{K}{a^2}, \end{aligned}$$

15 $$\begin{aligned}&\frac{\ddot{a}}{a}= -\, \frac{4\pi G}{3} \left( \rho +3P \right) , \end{aligned}$$

16 $$\begin{aligned}&\dot{\rho }+3H\left( P+\rho \right) =0, \end{aligned}$$

where *G* is Newton’s constant, units in which the speed of light is unity are used, differentiation with respect to the comoving time *t* is denoted by an overdot, and $H(t)\equiv \dot{a}/a$ is the Hubble function [9–13]. There are only two independent equations in the set (14)–(16) since any one of them can be derived from the other two. Without losing generality, we choose the Friedmann equation (14) and the energy conservation equation (16) as independent, then the acceleration equation (15) follows from them.

Equation (14) with $K=0$ is formally the same as the squared Omori differential equation (12) under the exchange $n(t) \longrightarrow a(t)$ provided that the analogous universe is sourced by a suitable cosmological fluid. Equations (14) and (12) considered jointly imply that it must be

17 $$\begin{aligned} \rho (t)= \rho _0 a^2(t) \,, \end{aligned}$$

where $\rho _0$ is a positive integration constant determined by the initial conditions and such that

18 $$\begin{aligned} \sigma ^2 = \frac{8\pi G \rho _0}{3} \,. \end{aligned}$$

In FLRW cosmology, where the cosmic fluid satisfies the barotropic equation of state $P=w\rho$, $w=$ const., Eq. (16) integrates immediately to

19 $$\begin{aligned} \rho (a) = \frac{ \rho _0}{ a^{3(w+1)} } \,. \end{aligned}$$

The corresponding solution of the Friedmann equation is

20 $$\begin{aligned} a(t)=\frac{a_0}{ \left| t-t_0\right| ^{3|w+1| } } \,. \end{aligned}$$

**Fig. 1:**
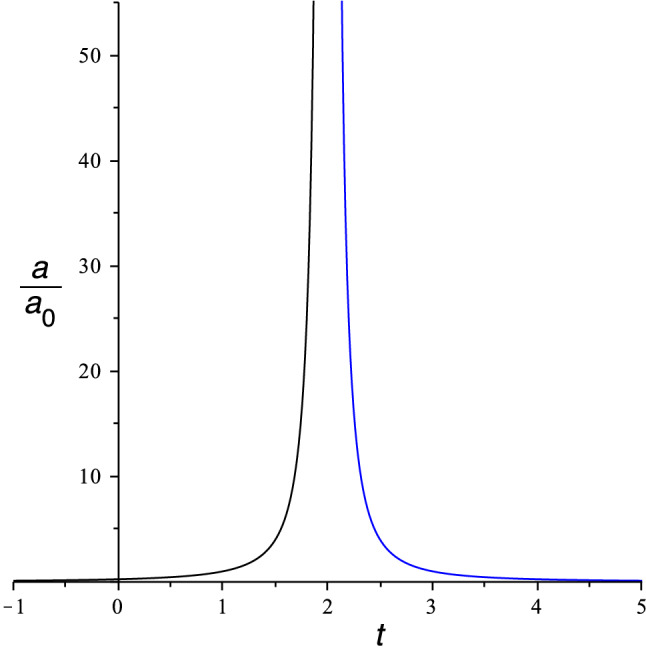
The scale factors of an expanding universe approaching the Big Rip and of a contracting universe emerging from it, for equation of state parameter $w=-5/3$ and $t_0=2$ in arbitrary units

The comparison of Eqs. (17) and (19) shows that the analogy between earthquakes and cosmology is valid if the universe is filled with a perfect fluid with $$P=w\rho $$ and equation of state parameter $$ w= -5/3$$ (Fig. 1).

The aftershock regime corresponds to a contracting universe with decreasing *a*(*t*) and $\dot{n}<0$, while the beforeshock phase corresponds to an expanding analogous universe and $\dot{n}>0$.

It is well known [9, 10] that the Friedmann equation is a first order constraint and not a truly dynamical (second order) equation of motion. This constraint (“Hamiltonian constraint”) corresponds to the vanishing of the Hamiltonian of general relativity [9, 10, 13], and this is exactly the role played by the law (2), as seen in Eq. (8). The facts that the Friedmann equation looks like an energy conservation equation for one-dimensional motion and that it can describe a variety of different universes makes it suitable for several analogies between the cosmos and unrelated physical systems, including Bose–Einstein condensates [14–19], glacial valleys [20–22], capillary fluids [23], equilibrium beach profiles [24], and freezing bodies of water [25].

In the aftershock phase with $\dot{n}<0$, the analogous Friedmann equation describes a spatially flat ($K=0$) contracting universe fueled by a perfect fluid with energy density $\rho =\rho _0 a^2$ and equation of state parameter $w= -5/3$. This “phantom fluid” violates all the energy conditions expected to hold for physically reasonable matter [9–12]. Nevertheless, phantom matter is the subject of a large body of literature in cosmology because it can potentially explain a superaccelerating (i.e., $\dot{H}>0$) universe often preferred by cosmological observations.

A peculiar feature of a phantom fluid is that it causes a universe filled with it to expand so fast that it explodes at a finite time in a Big Rip singularity [26, 27]. Contrary to the better known Big Bang or Big Crunch singularities where the scale factor vanishes, in a Big Rip *a*(*t*) diverges. Scalar curvature invariants, as well as the energy density $\rho$ and the pressure *P* also diverge, making the Big Rip a genuine spacetime singularity [26, 27].

In our analogy, $t = |c|$ corresponds to the main earthquake shock and is analogous to the Big Rip singularity, while the aftershock phase $\dot{n}<0$ corresponds to the less studied branch of a universe *contracting* from a Big Rip. The expanding and contracting branches on either side of the Big Rip are disconnected because a spacetime manifold stops at a curvature singularity (in this case, the Big Rip), which is not part of spacetime itself. The expanding branch of the phantom universe has an analog in the Omori law with sign changed, $\dot{n}=\sigma n^2$, which can be used to model the beforeshock phase of an earthquake, during which smaller shocks become more and more frequent and lead to the main shock [2, 3]. The main earthquake separating beforeshock and aftershock regimes is analogous to the Big Rip singularity.

## Discussion and conclusions

We have developed an analogy between Omori’s law for the aftershocks following a main earthquake event and a spatially flat universe in FLRW cosmology, which is sourced by a phantom fluid and contracting. It is natural to extend this analogy to include a beforeshock phase corresponding to an expanding universe sourced by the same (or another) phantom fluid. The Big Rip singularity separating the expanding and contracting universes is analogous to the spacetime singularity.

Formally, the catastrophic nature of the solution of Eq. (2) and of $\dot{a} \approx a^2$ is due the fact that the exponent 2 in the right hand side prevents the existence of a maximal solution defined on an infinite half-interval [33]. The analogy has some value for physical intuition. Indeed, the Lagrangian (4) for Omori’s law is derived using as an example the effective point-like Lagrangian for a FLRW universe sourced by a perfect fluid, which is (e.g., [28–32])

21 $$\begin{aligned} L\left( a, \dot{a}, P \right) = 3a\dot{a}^2 -a^3 P \,. \end{aligned}$$

Another consideration is in order. Aftershocks are often modeled with the generalized Omori (or Omori-Utsu) law [34, 35]

22 $$\begin{aligned} n(t) = \frac{k}{ \left( t-|c|\right) ^p}\,, \end{aligned}$$

where the exponent *p* varies according to the location and the specific earthquake in a rather wide range [36]. In this case the analog of Eq. (2) is

23 $$\begin{aligned} \dot{n} =-\frac{p}{k^p} \, n^{\frac{p+1}{p} } \equiv -\sigma _{(p)} n^{\frac{p+1}{p}} \,. \end{aligned}$$

One can generalize the previous reasoning for $p=1$: the Lagrangian is now

24 $$\begin{aligned} L_{(p)} \left( n, \dot{n}\right) = n \dot{n}^2 +\sigma _{(p)}^2 \, n^{\frac{3p+2}{p}} \,, \end{aligned}$$

the second order equation of motion is

25 $$\begin{aligned} 2n\ddot{n}+\dot{n}^2 -\frac{(3p+2)}{p} \, \sigma _{(p)}^2 \, n^{ \frac{2(p+1)}{p}} \,, \end{aligned}$$

while the Hamiltonian is

26 $$\begin{aligned} {\mathcal {H}}_{(p)}= n \left( \dot{n}^2 - \sigma _{(p)}^2 \, n^{\frac{2(p+1)}{p}} \right) \,; \end{aligned}$$

it is conserved, and its value is again ${\mathcal {H}}_{(p)}=0$. The analogy with cosmology is still valid and, for the range of values of $p>0$ encountered in the literature, the cosmic fluid is again a phantom fluid with equation of state parameter

27 $$\begin{aligned} w_{(p)} = - \frac{(3p+2)}{3p} \end{aligned}$$

causing again a Big Rip (which always occurs for equation of state parameters $w<-1$ [26, 27]).

In principle, a deviation of the exponent *p* from unity ruins the simple derivation of Refs. [5–7]. These authors attribute deviations from the simple Omori law (1) to a time dependence of the coefficient $\sigma$ instead. In the cosmological analogy, a varying $\sigma$ corresponds to a time-varying gravitational constant *G* (cf. Eq. (18)), which is impossible in general relativity. Such a variation is an essential part of scalar-tensor cosmology, but this possibility necessarily implies the presence of additional terms in the Friedmann and acceleration equations (14) and (15) [32, 37–39]. The lesson from cosmology would be that the variation of $\sigma$ involves extra energy terms associated with $\dot{\sigma } \ne 0$ in an energy balance involving the variation of *n*. It is more natural, and common in the cosmological literature, to allow for a different equation of state parameter or, perhaps, for time-dependent equation of state of the cosmic fluid $P(t)=w(t) \rho (t)$. This would still be a perfect fluid and can be realized, for example, by a scalar field with a dynamical equation of state, as in early universe inflation [12, 13, 40–43] and the late time, dark energy-dominated, era [44]. Both procedures would imply the introduction of another element in the fundamental derivation of the Omori law of Refs. [5–7], perhaps a distribution of intersecting faults with more than two adjacent sides involved. Here we do not speculate further on this new element. In any case, the search for fundamental and universal laws as opposed to mere data-fitting lies at the core of science. Lagrangian and Hamiltonian formulations and analogies can perhaps help in the search for these laws.

## Data Availability

This manuscript has no associated data or the data will not be deposited. [Author’s comment: There are no data associated with this manuscript.]

## Footnotes

1 The Omori law is used also to describe seismicity rates before and after eruptions [2, 3].

2 In principle, however, the deactivation coefficient $\sigma$ could depend on time, introducing nonstationarity and deviations from a strict Omori law [5, 7].
